# Electrocutions – treatment strategy (case presentation)

**Published:** 2014

**Authors:** M Ungureanu

**Affiliations:** * “Grigore Alexandrescu” Hospital, Plastic and Reconstructive Surgery and Burns Department

**Keywords:** electrocution, escharotomy, fasciotomy, skin grafting

## Abstract

Electrical injuries are a form of trauma with extreme gravity and a unique pathophysiology: they affect the entire organism.

A wide range of voltages may cause electrical accidents. Complications should be anticipated and prevented in order to minimize the complication risk and assure a vital, functional and esthetic prognosis as good as possible.

The article presents a case treated in our clinic together with the unique particular clinical situation and algorithm that led to a favorable result.

## Introduction

Electrical injuries represent trauma with extreme gravity; they have a unique pathophysiology. They encompass several types, as it follows: lightning injury, high-voltage injury and low-voltage injury. The clinical manifestations range from transient unpleasant sensations without an apparent injury to massive tissue damage. Some electrocutions are instantly fatal. The familiarity with the mechanisms of injury and the principles of therapy improve patient care [**1**-**3**].

The 3 major mechanisms of electricity-induced injury are the following:

Electrical energy causing direct tissue damage, altering cell membrane resting potential, and eliciting muscle tetany.

Conversion of electrical energy into thermal energy, causing massive tissue destruction and coagulative necrosis. 

Mechanical injury with direct trauma resulting from falls or violent muscle contraction [**6**].

Factors that determine the degree of injury include the magnitude of energy delivered, resistance encountered, type of current, current pathway and duration of contact. The systemic effects and tissue damage are directly proportional to the magnitude of current delivered to the victim [**7**].

The electrical shock is classified as high voltage (>1000 volts) or low voltage (<1000 volts). As a rule, high voltage is associated with greater morbidity and mortality, although fatal injury can occur at household current (110 volts). The current pathway determines which tissues are at risk and the type of injury that is observed. Electrical current that passes through the head or thorax is more likely to produce a fatal injury. Transthoracic currents can cause fatal arrhythmia, direct cardiac damage, or respiratory arrest. Transcranial currents can cause direct brain injury, seizure, respiratory arrest, and paralysis [**9**,**10**,**12**].

Electrothermal tissue injury results in tissue edema; therefore, the development of a compartment syndrome can occur in any body compartment. The leg is the site most commonly involved in the development of compartment syndrome.

Clinical presentations range from a tingling sensation to a widespread tissue damage and even to instantaneous death.

All the circuits may produce myonecrosis, myoglobinemia, and myoglobinuria and their attendant complications.

Circuits may produce electrical burns with relatively massive amounts of tissue destruction by heating the tissues (physical property of friction from the passages of electrons [joule heating]) and by the destruction of cell membranes by producing holes in the membranes (poration).

In addition, thermal burns resulting from the electrical flashes are generally considered electrical injuries, although such injuries may not involve a circuit through a person.

There is a variety of factors that can affect the severity of the injury. With high-voltage injuries, most of the injuries appear to be thermal and most histologic studies reveal coagulation necrosis consistent with the thermal injury. Lee and others have proposed the theory of electroporation in which the electrical charges are too small to produce thermal damage, cause protein configuration changes threatening cell wall integrity and cellular function. Some believe that magnetic effects may also be on the tissue. Nerves designed to carry electrical signals, and muscle and blood vessels, because of their high electrolyte and water content, are good conductors. Bone, tendon, and fat have a very high resistance and tend to heat up and coagulate rather than transmit current [**11**]. The other tissues of the body are intermediate in resistance. Skin is the primary resistor to the flow of current into the body. Much of the energy may be dissipated at the skin surface, causing significant surface burns in a heavily calloused area, sometimes resulting in less deep internal damage than it would be expected if the current were delivered undiminished to the deep tissues [**4**,**5**,**8**].

In general, the longer the duration of the contact with high voltage current, the greater the degree of tissue destruction [**14**].

High voltage has a greater potential for tissue destruction and can be responsible for severe injuries leading to major amputations and tissue loss [**14**].

The pathway that a current takes determines the tissues at risk, the type of injury seen, and the degree of conversion of electrical energy to heat regardless of whether high, low, or lightning voltages are being considered. Current passing through the heart or thorax can cause cardiac arrhythmias and direct myocardial damage. The current passing through the brain can result in respiratory arrest seizures, direct brain injury and paralysis. Current passing close to the eyes can cause cataracts [**13**].

As current density increases, its tendency to flow through the less-resistant tissues is overcome, so that it eventually flows through the tissues indiscriminately, treating the body as a volume conductor, with potential destruction of all tissues in the current's path. Damage to the internal structures of the body may be irregular, with areas of normal-appearing tissue next to the burnt tissue and with damage to structures at sites distant from the apparent contact and ground points [**1**-**3**].

Probably, the most important difference between light- and high-voltage electrical injuries is the duration of exposure to the current [**1**-**3**].

It is often difficult to determine which mechanism of injury has caused burns at the time of a patient's presentation to the emergency department. This may make it difficult to assess the injury and offer a prognosis based on history and physical examination alone. The most destructive indirect injury occurs when a person becomes part of an electrical arc, since the temperature of an electrical arc is of approximately 2500 degrees Celsius. The arc may cause clothing to ignite and cause secondary thermal burns. The electrical flash burn usually results in only superficial partial-thickness burns [**16**,**17**].

Blunt injury may occur in electrical injury as the person is thrown clear of the source.

Often, the diagnosis certainty is given by electrocution marks.

The development of increased myofascial compartment pressures is of great concern. If this is suspected, each compartment must be measured. If signs and symptoms of compartment syndrome exist, decompression is necessary. The hallmark of compartment syndrome is pain with passive motion in the compartment containing the muscle groups responsible for that motion. Characteristically, the pain is unrelenting and may appear out of proportion to the visible injury. Patients may experience paresthesia, hypoesthesia, or decreased motor function. Loss of pulses is actually a late sign of compartment syndrome [**15**].

Electrical injuries are usually self-evident from history and physical surroundings, except for the case of bathtub accidents, where no burns occur, or of foul play. It is necessary to attempt to differentiate between mechanisms of burn injury because flash burns have a much better prognosis than arc or conductive burns. Injuries from blunt trauma and falls may also be present. Electrocutions with tegument lesions over 30% TBSA or with associated lesions often present extreme gravity.

## Material and methods

A case of electrocution treated in our clinic is depicted. The patient is a 13-year-old male.

On admission, his general state was of extreme gravity with postcombustional shock after having suffered electrocution – railway voltage. Tegument lesions encompassed approximately 80% TBSA, mainly deep partial and full thickness.

The patient was initially evaluated as a trauma patient. Airway, breathing, circulation were performed as a part of primary survey. Intravenous access, cardiac monitoring, and measurement of oxygen saturation were started during the primary survey. Fluid replacement was the most important aspect of the initial resuscitation.

Local debridement was performed and reanimation protocol continued.

On admission, the tegument lesions were presented as:

- full thickness burns localized on: left arm, left trunk anterior and posterior – approximately 20% TBSA

- deep partial burns localized on: anterior trunk, posterior trunk, anterior cervical, right upper limb, thighs and legs – approximately 50% TBSA

- superficial partial burns localized on: face, upper and lower limbs – approximately 10% TBSA.

Escharotomy and fasciotomy were performed in the left upper limb and trunk relieving compartment pressure in left upper limb and improving ventilation. Fasciotomy had a dual role as both a therapeutic tool and a diagnostic tool in the treatment of electrical injuries. Deeper structures lesions were ruled out, considering the fact that a burn with a relatively small surface area may hide massive tissue destruction beneath.

**Fig. 1:**
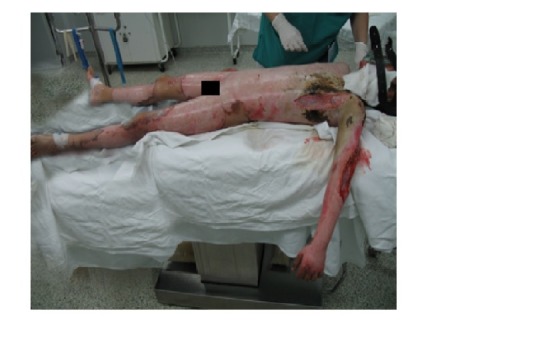
Surgical treatment in acute phase – escharotomy and fasciotomy, left anterior lateral thorax and left upper limb

On the first day after admission especially and all throughout admission, the patient was subject to special intensive care monitoring.

The extent or volume of tissue damage involved with an electrical injury was difficult to assess.

Galverstone Carvajal formula supplemented was used to estimate the initial necessary resuscitation volume over the first 24 hours.

The patient was unable to give a good history because of the severity of injury and the accompanying shock and hypoxia. Resuscitative efforts continued in the emergency department with adequate fluid administration and insertion of a Foley catheter. No prediction regarding the exact amount of the underlying tissue damage from the amount of cutaneous involvement could be made and so fluid resuscitation was supplemented according to protocol. A urine output of at least 1.5 ml/kg/hr was maintained.

Early physical and occupational therapy reduced limbs dysfunction.

Because the majority of the wounds presented as deep partial burns, the choice to avoid the initial excision and grafting and perform a proper reanimation and meticulous local treatment was made. Furthermore, there was no evident limit between IIB and III degree lesions.

Antiseptic dressings with silver sulfadiazine were applied twice a day after sterilizing the wounds with betadine and chlorhexidine.

Good quality reanimation was preferred, earning 30% epithelization and 30% in course of epithelization, while only 12% of the full thickness lesion of the initial lesions was grafted.

**Fig. 2:**
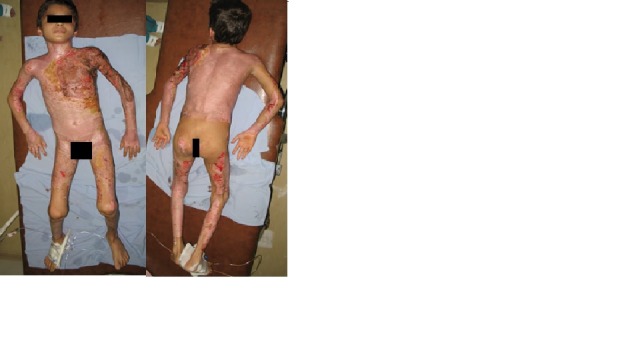
Local aspect after 25 days from admission – integrated autografts on the anterior thorax and left upper limb; spontaneous epithelization on the remaining areas of the initial burn lesions

The excision and grafting was performed after 16 days so by day 28 he was quite completely covered (grafts and epithelization).

No early excision was performed because partial deep and full thickness burns were not well delimited and the percent of full thickness burns was reduced.

## Results

The results were remarkable. We managed to epithelize most of the deep partial wound after 2 weeks of treatment.

On the 17th day after accident, eschars suprafascial excision was performed (left trunk and left arm, approximately 12% T.B.S.A.) under general anesthesia and the covering with skin grafts harvested from the inferior limbs was performed. The surgical intervention practically removed the remaining eschar and the skin grafts took up the following week.

The local evolution was favorable with graft integration and donor zone epithelization. Three weeks after the accident, the patient was transferred to a local hospital with a good general state, cardio-vascular, respiratory, digestive equilibrated, afebrile.

According to general state, throughout hospitalization, the patient benefited from kinesiotherapy rehabilitation for grafted segments functionality.

After less than a month treatment, the patient was transferred to the local hospital, with initial lesions epithelized in a proportion of 95%.

## Conclusion

Electrocution burns constitute a disease with predictable evolution and adequate treatment of re-equilibration and surgical interventions. They can have favorable results even on large burnt surfaces.

This case was seen as an example of shock exit and a particular strategy for a patient with extensive burns after a major electrocution.

This case was approached in an original way trying to assure the best healing conditions for partial deep burns representing most of the total injury.
